# Acute Caudate Nucleus Stroke Presenting As Hemiballismus

**DOI:** 10.7759/cureus.48209

**Published:** 2023-11-03

**Authors:** Denis Babici, Ali A Mohamed, Olivia Mattner, Francis Demiraj, Thomas Hammond

**Affiliations:** 1 Department of Neurology, Florida Atlantic University Charles E. Schmidt College of Medicine, Boca Raton, USA; 2 Department of Medicine, Florida Atlantic University Charles E. Schmidt College of Medicine, Boca Raton, USA

**Keywords:** subthalamic nucleus, haloperidol, basal ganglia stroke, basal ganglia infarction, chorea and hemiballismus

## Abstract

Hemiballismus is defined as irregular, involuntary, large-amplitude flinging movements by the limbs, confined to one side of the body. Hemichorea refers to a state of excessive and irregularly timed, non-repetitive and randomly distributed, spontaneous, involuntary, and abrupt movements. It is widely believed that hemiballismus and chorea are suggestive of a lesion to the basal ganglia and subthalamic nucleus (STN). However, there are other etiologies that may influence the clinical presentation. Patients may present with certain common clinical features corresponding to the affected area of the brain. For example, infarctions of the motor cortex present with hemiplegia or paralysis of one side of the body. Similarly, infarctions involving the language areas of the brain present with aphasia and are detrimental to speech production or comprehension and the ability to read and write. Typically, acute-onset hemichorea is suggestive of a lesion in the STN. Herein, we present a rare case of acute hemiballismus and hemichorea following infarction of the left caudate nucleus, as determined by magnetic resonance imaging (MRI) and computerized tomography (CT) imaging modalities.

## Introduction

Chorea is a unilaterally presenting involuntary movement disorder described as sudden, brief, spontaneous, non-stereotyped, and dance-like. Classically, the literature establishes chorea as the result of a lesion to the subthalamic nucleus (STN) or the nucleus hypothalamus/corpus Luysi. However, other investigations have identified localized lesions in other parts of the basal ganglia and caudate nucleus as the culprits of the movement disorder [1]. Despite uncertainty regarding the prevalence and incidence of chorea syndromes, it is estimated that one to two individuals are affected per 1,000,000 [2]. Although many types exist, hemiballismus is recognized as the most severe form of chorea. Hemiballismus is an involuntary, hyperkinetic movement disorder caused by central nervous system dysfunction on the contralateral side. Hemiballismus presents as high-amplitude movements of the ipsilateral upper and lower extremities. Common descriptions include movements that are flinging, violent, ballistic, sudden, involuntary, and/or intermittent [2]. The hyperkinetic movement disorder theory presents a more comprehensive pathophysiological mechanism that requires discussion of the complex basal ganglia inhibitory and excitatory pathways. The planned activation and inhibition of movement are generated by cortex input that is transmitted to the nuclei of the basal ganglia. These nuclei include the caudate, putamen, globus pallidus (constituting the globus pallidus internus and externus), STN, and substantia nigra. In the absence of pathology, basal ganglia nuclei project subsequent output fibers that convert into the lenticular fasciculus tract. This white matter tract forms a bundle called the thalamic fasciculus that then enters the thalamus. Within the thalamus, excitatory and somatosensory signals are relayed to the motor cortex. Motor neurons within the frontal lobe and brainstem then initiate or terminate movement.

Chorea is a consequence of damage to the inhibitory pathways within the basal ganglia. Normally, the globus pallidus internus (GPi) inhibits the thalamus through inhibitory projections and thus modulates the movement pathways. However, when there is a decrease in the inhibitory transmission from the GPi to the thalamus, the thalamus starts randomly firing excitatory projections that overactivate the corticospinal and corticobulbar tracts. The resulting efferent innervation transmitted to the muscles on the contralateral side creates the abnormal movements described as chorea.

The vascular supply of the basal ganglia is also a key component of chorea’s presenting pathology. Overall, the basal ganglia are supplied by a mix of small feeder vessels stemming from the anterior cerebral, middle cerebral, posterior cerebral, and posterior communicating arteries. The lateral lenticulostriate artery stems from the middle cerebral artery and supplies portions of the putamen, external capsule, and upper internal capsule. The anterior cerebral artery feeds the medial lenticulostriate artery and the medial striate artery of Huebner, which supply the globus pallidus and portions of the caudate, respectively. The caudate does receive some supply from the middle cerebral artery as well [3]. The posterior cerebral and posterior communicating arteries supply the substantia nigra and STN [4].

Like any blood vessel in the brain, the vascular supply to the basal ganglia is at risk of damage from strokes. Strokes causing hemichorea-hemiballismus usually localize to a common functional network, with lesions occurring in regions functionally connected to the posterolateral putamen. To determine treatment efficacy, the neurology-neurosurgical team assesses perioperative neuronal activity. Monitoring studies have demonstrated decreased firing rates of cells within the GPi following pallidotomy, a surgical treatment aimed at reducing hyperkinetic movements. Considering the complex pathophysiology, hemiballismus is suggestive of a lesion to the basal ganglia STN. However, rare cases have demonstrated other etiologies to be responsible, with different etiologies having unique clinical features of their own. Herein, we present a rare case of hemiballismus following a stroke of the left caudate nucleus.

## Case presentation

**Figure 1:**
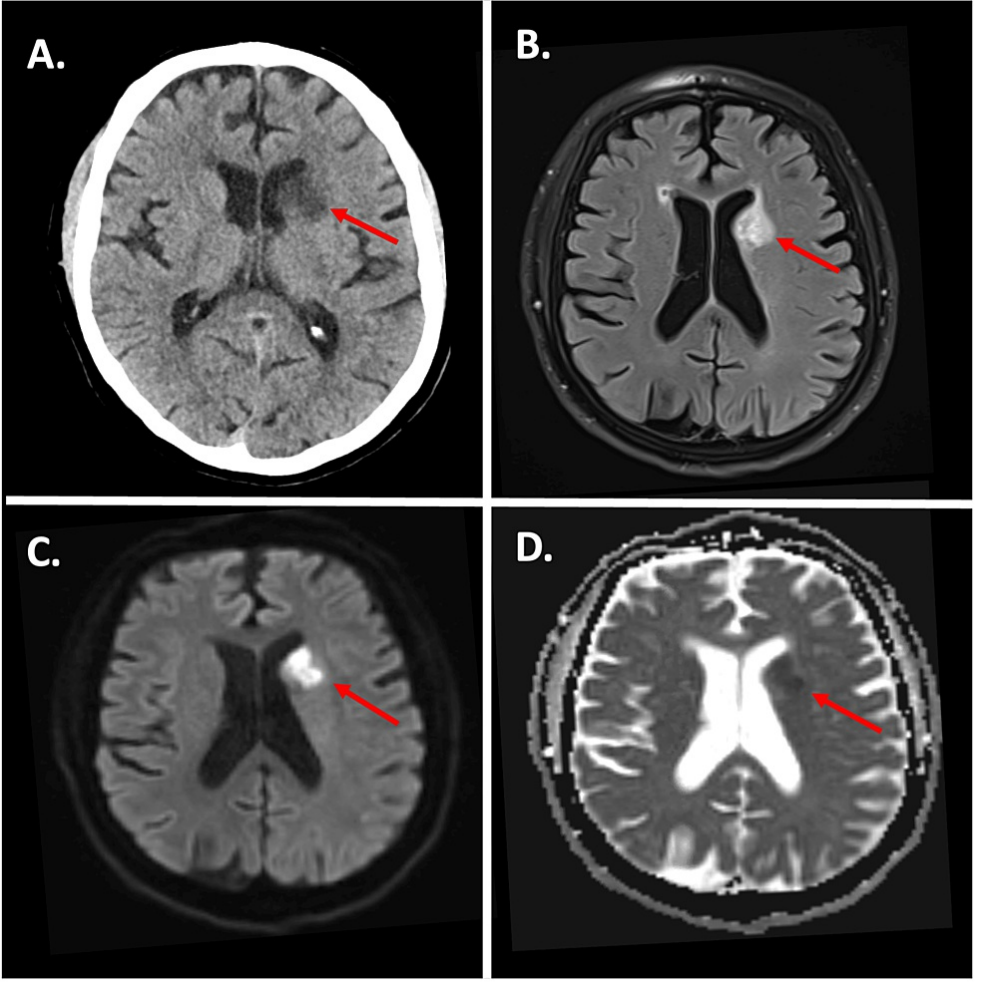
CT and MRI following left caudate nucleus stroke A. CT head showing hypodensity in the left basal ganglia; B. FLAIR sequence of MRI showing hyperintensity in the left caudate nucleus; C and D. DWI and ADC sequences of MRI showing restricted diffusion in the left caudate nucleus (red arrows). FLAIR: fluid-attenuated inversion recovery; DWI: diffusion-weighted imaging; ADC: apparent diffusion coefficient

A 64-year-old male with a medical history of a non-disabling stroke, tobacco use, and alcohol use disorder was admitted to the hospital due to a four-day history of right-sided weakness and involuntary shaking movements of his right arm and leg. He reported not feeling well and falling to the right with attempts to walk. The patient’s wife reported noticing involuntary choreiform movements in the patient’s right hand and foot and occasional arm-flinging, ballistic-like movements two days prior to admission to the hospital. A computerized tomography (CT) scan of the head revealed hypodensity involving the left caudate nucleus, consistent with an infarction (Figure 1A).

**Figure 2:**
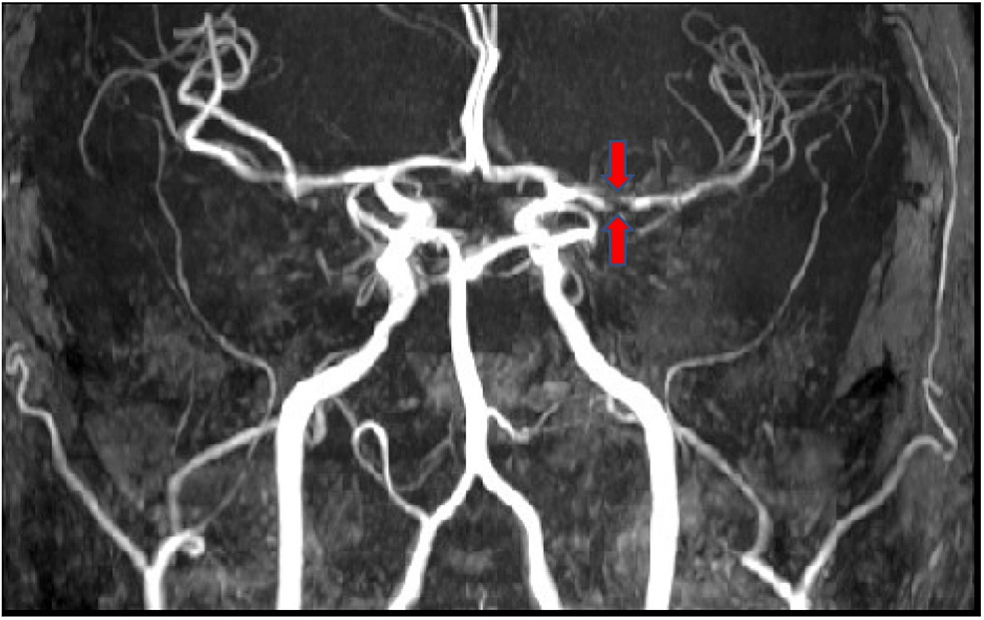
MRA of the head following left caudate nucleus stroke Severe stenosis of the left M1 segment of the middle cerebral artery (red arrows) MRA: magnetic resonance angiography

Magnetic resonance imaging (MRI) of the brain was positive for an acute stroke of the left caudate nucleus with restricted diffusion and signal loss on the apparent diffusion coefficient (ADC) map (Figures 1B-1D). Magnetic resonance angiography (MRA) of the head was significant for severe left middle cerebral artery (MCA) stenosis (Figure 2).

Over the next few days, the right-sided weakness improved gradually, but the frequency of choreiform movements increased. The patient was started on low-dose haloperidol, which significantly improved his hemichorea symptoms.

## Discussion

Hemichorea-hemiballismus is uncommonly reported following a stroke. The incidence of this hyperkinetic movement disorder following an acute ischemic stroke ranges between 0.4% and 0.54% [5, 6]. These characteristic movements occur as a consequence of disruption to the complex network formed between the basal ganglia and various distinct regions of the brain [6]. Hemichorea-hemiballismus is classically associated with damage to the STN but more commonly occurs following damage to the contralateral striatum [5]. Other causative lesion sites include the thalamus, the posterior limb of the internal capsule, the external capsule, corona radiata, pons, and the frontal and parietal cortices [7].

The basal ganglia are modulators of motor control. Damage to these basal ganglia structures will lead to hyperkinetic or bradykinetic states. For example, loss of cells in the substancia nigra leads to parkinsonism with bradykinesia. Lesions involving the STN are felt to result in hyperkinetic states, chorea, and/or hemiballismus. Similarly, lesions of the medial globus pallidus increase motor activity (a reason for the benefit of pallidotomy procedures for Parkinson's disease in years past). Acute-onset hemichorea has been felt to be caused by a subthalmic stroke [5]. However, our case demonstrates that this phenomenon can occur with an acute stroke involving the caudate nucleus.

Given the caudate’s mixed vascular supply of the anterior and middle cerebral arteries, it normally would be difficult to ascertain which vessel caused a presenting ischemic infarct. Fortunately, in our case, the severe stenosis of the left M1 of the middle cerebral artery leads us to believe that this was the causative vessel. The middle cerebral artery gives rise to the lenticulostriate perforates, which supply the superior head and body of the caudate and suggest possible localized areas of infarction. One caveat for individuals suffering from high levels of intracranial stenosis is the possibility of contralateral supply to the caudate nucleus [8].

Medical management aims to relieve severe hyperkinetic movements using first- and second-generation antidopaminergic drugs that target dopamine receptors (D2Rs) (e.g., risperidone, haloperidol, perphenazine, pimozide, and chlorpromazine), benzodiazepines (clonazepam), anti-epileptics (topiramate), and tetrabenazine [9-11]. Hemiballismus that is responsive to treatment typically has a favorable prognosis, demonstrating remission of symptoms within a few weeks of treatment.

In the discussed case, the patient presented with multiple stroke risk factors and was found to be positive for stroke on MRI. Based on the patient’s medical history and MRI findings, a lab test was not required [2]. The patient had an excellent response to medical management with low-dose haloperidol, demonstrating significant improvement in hyperkinetic movements.

## Conclusions

When assessing patients presenting with involuntary movements in the acute stroke setting, identification of the dysfunctional area of the brain can be a challenge. In this setting, regular and rhythmic movements suggest focal-onset seizures, whereas irregular hemichorea or hemiballismus movements suggest disruption of deep basal ganglia motor control regions. In this instance, neuroimaging is an important tool for establishing a definitive and accurate diagnosis. Furthermore, imaging studies can help expand the current understanding of the impacts of the dysfunction of different brain regions.

In the presented case, a patient with hemiballismus and hemichorea following an infarction of the caudate nucleus was identified by CT and MRI imaging modalities. This case highlights the value of diagnostic imaging and the potential for variable anatomic regions to be involved in the pathophysiology of involuntary movement disorders. Although hemichorea and hemiballismus are believed to be consequences of strokes involving the STN, this case demonstrates that infarction of other basal ganglia brain structures, specifically the caudate nucleus, may be responsible for this rare clinical stroke presentation.
